# Animal- and Human-Inspired Nanostructures as Supercapacitor Electrode Materials: A Review

**DOI:** 10.1007/s40820-022-00944-z

**Published:** 2022-10-06

**Authors:** Iftikhar Hussain, Charmaine Lamiel, Sumanta Sahoo, Muhammad Sufyan Javed, Muhammad Ahmad, Xi Chen, Shuai Gu, Ning Qin, Mohammed A. Assiri, Kaili Zhang

**Affiliations:** 1 Department of Mechanical Engineering, City University of Hong Kong, 83 Tat Chee Avenue, Kowloon, Hong Kong People’s Republic of China; 2 Department of Chemical Engineering, University of Wyoming, Laramie, WY 82071 USA; 3 Department of Chemistry, Madanapalle Institute of Technology and Science, Madanapalle, Andhra Pradesh 517325 India; 4 School of Physical Science and Technology, Lanzhou University, Lanzhou, 730000 People’s Republic of China; 5 Department of Chemistry, Faculty of Science, King Khalid University, Abha, 61413 Saudi Arabia

**Keywords:** Nature-inspired nanostructure, Supercapacitors, Energy storage, Animal-inspired and human-inspired nanostructures

## Abstract

Human civilization has been relentlessly inspired by the nurturing lessons; nature is teaching us. From birds to airplanes and bullet trains, nature gave us a lot of perspective in aiding the progress and development of countless industries, inventions, transportation, and many more. Not only that nature inspired us in such technological advances but also, nature stimulated the advancement of micro- and nanostructures. Nature-inspired nanoarchitectures have been considered a favorable structure in electrode materials for a wide range of applications. It offers various positive attributes, especially in energy storage applications, such as the formation of hierarchical two-dimensional and three-dimensional interconnected networked structures that benefit the electrodes in terms of high surface area, high porosity and rich surface textural features, and eventually, delivering high capacity and outstanding overall material stability. In this review, we comprehensively assessed and compiled the recent advances in various nature-inspired based on animal- and human-inspired nanostructures used for supercapacitors. This comprehensive review will help researchers to accommodate nature-inspired nanostructures in industrializing energy storage and many other applications.

## Highlights

- Animal- and human-inspired nanostructures as supercapacitor electrode materials are summarized.
- Structural formation and supercapacitive electrochemical applications are comprehensively summarized.
- Future outlooks such as large-scale production and other properties are proposed.

## Graphical abstract

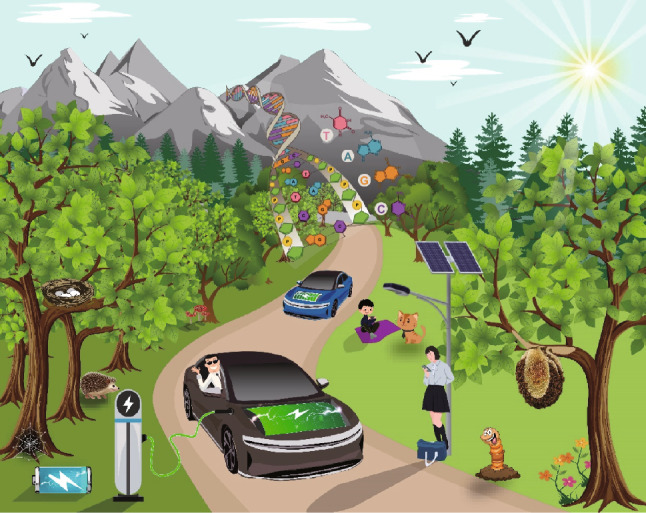

## Introduction

Over the years, nature plays an important role in the development of mankind. Many revolutionary discoveries of today’s world have been inspired by nature. In the area of materials science, such inspiration from nature has been successively employed in fabrication processes as well as to design target materials. The highly ordered, diverse, and unique structures of natural things and biomaterials inspired researchers to duplicate and mimic it in nanomaterials through material chemistry [1–7]. The inspiration from nature and the designing of nanomaterials with appropriate orientation, highly ordered structure, and exceptional mechanical robustness while achieving high energy and power densities have remained a hot topic in developing electrode materials. Many nature-inspired commercial products are also easily available in today’s commercial market [8]. For example, artificial photosynthesis, inspired by the photosynthesis of plant, approach has been successively employed for harvesting solar energy [9, 10]. Other nature-inspired applications include bio-inspired water purification system [11], protein production inspired from the silk making process of spiders [12], bio-inspired materials for plastic replacement [13], etc. have also been reported. Moreover, nature-inspired materials and designs have also been explored to produce natural and renewable resources toward sustainable, low-cost electrode development [14– 17].

The enthusiastic development of cutting-edge energy storage devices advances today’s electronics world for the betterment of tomorrow. Supercapacitors (SCs) are one of such elite electrochemical energy storage devices which have gained enormous research interest in last few years [18–27]. Specially, the charge storage mechanism of electric double layer capacitor (EDLC) does not involve any chemical reaction [28– 30], which plays a crucial role for the designing of sustainable future. Benefitted by their enhanced charge storage mechanism, improved cycling stability, high rate capability, and elevated power density, SCs have shown promising advantages to fulfill the demand of future electronic devices [31– 38]. In this aspect, several strategies have been employed for enhancing the capacitive performance of SC electrodes. Among them, high porosity, high conductivity, and large surface area are some of the prime factors being considered [39– 42]. Basically, the porous nature of electrode materials allows easy and fast transport of electrolyte ions which further improves the electrochemical performance [43– 49]. While these properties are important, the morphology of the electrode materials is equally significant too [50–55]. The availability of large electrochemical surface area also relieves the stress incurred by long charging/discharging cycles. Among different morphologies, hierarchical morphologies are advantageous toward energy storage application due to their enhanced surface area, low density, controlled, interconnected structure, and enhanced accessible area. Such materials produce electrical, chemical, biological, mechanical, as well as sustainable gains, which are valuable toward the new developments in the energy-related fields [8]. In this aspect, nature-inspired materials with hierarchical structures are highly beneficial, such as, carbon materials with nature-inspired structures displaying high surface area and enhanced porosity. On the other hand, some metal oxides/mixed metal oxides also exhibited promising electrochemical characteristics, benefitted by their special nanoarchitectures [56– 59]. Although the nature-inspired materials are long way to go for commercialization, the current research trend is very encouraging for sustainable future. Despite of their impressive characteristics, very few systematic review articles on energy-related applications of nature-inspired materials are available [1, 6, 7, 60, 61]. Therefore, a timely update on such materials in this particular research field is highly necessary.

**Fig. 1:**
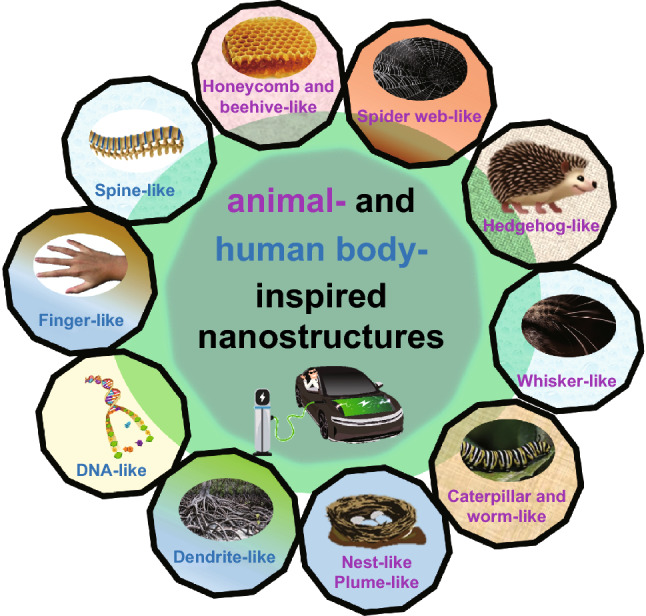
Schematic illustration of animal- and human body-inspired nanostructures

The aim of this paper is to summarize the applications of nature-inspired (animal- and human body-inspired) morphologies comprehensively for SC as shown in Fig. 1. Categorizing nature-inspired nanostructures according to a group of animal-inspired (honeycomb-, beehive-, spider web-, hedgehog-, whisker-, caterpillar and worm-, nest-, and plume-like) and human body-inspired (spine-, finger-, DNA-, and dendrite-like) nanostructures. This review discusses different factors and certain conditions capable of generating nature-inspired nanostructures. Moreover, the review ends with incorporating the learning from nature-inspired nanostructures with outlook, prospects, and strategies to mitigate the shortcomings of future electrodes in SC and battery applications.

## Nature-Inspired Structures: Synthesis, Structural Formation, and Supercapacitive Electrochemical System Application

As categorized in the dimensional growth of structures, the formation of 0D, 1D, 2D, and 3D structures are widely investigated in the mechanistic formation of crystals and their morphology [62–66]. Tiwari et al. [67] categorized different nanostructured materials as 0D, such as uniform particles arrays (quantum dots), heterogeneous particles arrays, core–shell quantum dots, onions, hollow spheres, and nanolenses; 1D such as nanowires, nanorods, nanotubes, nanobelts, and nanoribbons; 2D such as junctions (continuous islands), branched structures, nanoprisms, nanoplates, nanosheets, nanowalls, and nanodisks; and 3D such as nanoballs (dendritic structures), nanocoils, nanocones, nanopillars, and nanoflowers.

Even though these structures are classified into dimensional orientations, many studies have shown nanostructures mimicking things that we regularly see in nature. The overlapping and combination of 1D, 2D, and 3D nanostructures that resemble trees, honeycombs, flowers, urchins, etc., have been synthesized and worked effectively as an electrode material for SCs. Depending on many factors, such as choice of precursors, method of synthesis, the structure of an active electrode material can be customized in a way that more surface area can be exposed [53– 55, [68]. For example, carbon with tailored structure can achieve high surface area by engineering its morphology [69– 72] (Table 1). Such engineering of structures paved way for researchers to continuously study and evaluate hierarchical structures in a nanoscale level [73– 75].

**Table 1:** Specific surface area of carbon with various animal- and human-inspired structures

Nature-inspired structure	Electrode material	Specific surface area (m^2^ g^−1^)	Refs.
Beehive-like	Porous carbon	1472	[76]
Beehive-like	Microporous carbon	1327	[77]
Beehive-like	Porous carbon	1615	[78]
Honeycomb-like	Graphene	1962	[79]
Honeycomb-like	activated carbon	2990	[80]
Whisker-like	N-doped hollow porous carbons	3007	[81]
Worm-like	Nitrogen, sulfur-co-doped hierarchical porous carbon	720	[82]
Nest-like	N- and P-co-doped mesoporous carbon	922	[83]
Spine-like	Nanostructured carbon interconnected by graphene	428	[84]

In this section, we discuss the recent morphologies reported that imitate various nature-inspired such as animal and human body-inspired nanostructures, their method of synthesis, and their electrochemical properties applied as SC. This section is divided into groups according to animal-inspired (honeycomb-, beehive-, spider web-, hedgehog-, whisker-, caterpillar and worm-, nest-, and plume-like) and human body-inspired (spine-, finger-, DNA-, and dendrite-like) nanostructures.

### Animal-Inspired Structures

#### Honeycomb-Like Structure

With a similar structure, honeycomb and beehive-like structures have uniform and regularly shaped pores. Bio-inspired honeycomb-like [85–92] and beehive-like [76, 93, 94] structures with vertical thin walls have been greatly studied for SCs due to its excellent mechanical properties as well as exceptional active sites [2, 92, [95– 99]. Aside from SCs, bio-inspired honeycomb structures have also inspired further applications in biomedicine such as tissue engineering and regenerative medicine [97].

**Fig. 2:**
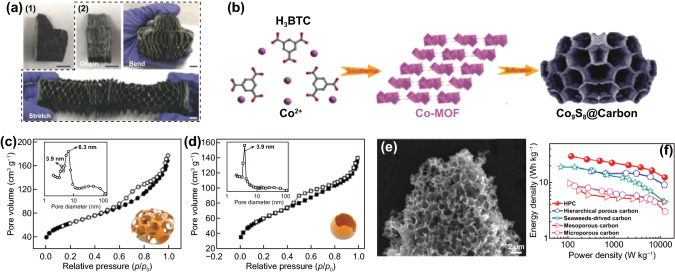
**a** 3D stretchable supercapacitors with various shape. Reproduced with permission from Ref. [100]. Copyright 2018, Wiley–VCH; **b** Schematic illustration of Co_9_S_8_@C‐500 synthesis strategy. Reproduced with permission from Ref. [101]. Copyright 2018, Wiley–VCH; N_2_ adsorption (close symbol)–desorption (open symbol) isotherms of RuO_2_·xH_2_O 3D architectures **c** RHCs and **d** RHSs; inset shows the corresponding BJH pore size distribution curves obtained from the desorption branch. Reproduced with permission from Ref. [102]. Copyright 2017, ACS Publications; **e** SEM image of HPC, **f** Ragone plots of the HPC symmetrical supercapacitor Reproduced with permission from Ref. [99]. Copyright 2015, Elsevier

Lv et al. [100] reported novel honeycomb-lantern-inspired 3D flexible and stretchable SCs for improved capacitance. The interesting structural flexibility and stretchability in shape of honeycomb-like structure offer mechanical strength as shown in Fig. 2a. The honeycomb-lantern-inspired structure was synthesized based on expandable composite electrode composed of polypyrrole/black-phosphorous oxide electrodeposited on carbon nanotube (CNT) film. The 3D honeycomb-lantern-inspired exhibited enhanced stretchability compared to 2D counterparts, which is useful for wearable devices. More importantly, the device is feasible to lessen the stress from different directions. The 3D SC maintained a capacitance of 95% under the reversible strain of 2000% even after 10,000 stretch and release cycles. Sun et al. [101] reported the metal–organic frameworks (MOFs) as a sacrificial template to prepared honeycomb-like metal sulfide as a SC electrode. Among different electrode materials, the MOF-derived honeycomb-like metal sulfide at 500 °C (Co_9_S_8_@C‐500) exhibited superior performance due to enhanced active sites of porous carbon thin nanosheets (Fig. 2b), which suppressed the agglomeration of metal sulfide and the improved conductivity of the electrode material due to carbon nanosheets. The Co_9_S_8_@C‐500 exhibited no fade in capacitance for 4000 cycles, confirming its excellent mechanical properties. Peng et al. [102] compared dynamic hydrolysis and static deposition approaches for the fabrication of SC electrode materials. Ruthenium oxide hollow sphere (static deposition) and honeycomb-like (dynamic hydrolysis) nanostructure were compared. The ruthenium oxide honeycomb-like (RHCs) electrode exhibited superior surface area (226 m^2^ g^–1^) than ruthenium oxide hollow sphere (RHSs)-like (226 m^2^ g^–1^) structure (Fig. 2c-d). Furthermore, the honeycomb-like electrode exhibited 5% higher cycling stability for the same number of cycles. Wu et al. [99] carbonized KOH-treated wheat flour in a single step to obtain 3D honeycomb-like porous carbon (HPC) foam nanostructure. The interconnected HPC (Fig. 2e) electrode material exhibited high surface area and was used for symmetric supercapacitor (SSC) device. The SSC device exhibited superior energy and power densities than reported carbon-based SSC devices as shown in Fig. 2f [77, 99, 103, 104]. In addition to that, various number of honeycomb-like structure have been reported for SC applications, confirming the importance of unique structure for energy storage applications. Table 2 shows the reported honeycomb-like structures.

**Table 2:** Comparison of animal-inspired structures in three-electrode measurements

Electrode structure	Electrode materials	Method	Three-electrode measurements			Refs.
			Capacitance	Cycling Stability	Electrolyte	
*Animal-inspired structures*						
*Honeycomb and beehive-like structures*						
Honeycomb	N-doped porous carbon	Carbonization	275 F g^–1^ 0.5 A g^–1^	99% 5,000 cycles 1 A g^–1^	6 M KOH	[143]
Honeycomb	FeMoO_4_ on NF	CBD	158.39 mA h g^–1^ 2 A g^–1^	90.76% 4,000 cycles 6 A g^–1^	3 M KOH	[92]
Honeycomb	Porous carbon	Carbonization	349 F g^–1^ 1 A g^–1^	98.6% 10,000 cycles 200 mV s^–1^	6 M KOH	[144]
Honeycomb	NiO	Hydrothermal	1,250 F g^–1^ 1 A g^–1^	88.4% 3,500 cycles 5 A g^–1^	6 M KOH	[145]
Honeycomb	Porous carbon	Hydrothermal, carbonization	227 F g^–1^ 1.5 mA cm^−2^	100% 2,000 cycles 40 mA cm^−2^	2 M KOH	[146]
honeycomb	NiCo_2_O_4_ on NF	Combustion method	646.6 F g^–1^ 1 A g^–1^	NA	6 M KOH	[98]
Honeycomb	O, N- carbon	Ethanol extraction, chemical activation	381 F g^–1^ 1 A g^–1^	NA	6 M KOH	[147]
			160 F g–1 1 A g–1	NA	1 M KOH	
honeycomb	Mo-ZnS@NF	hydrothermal	2,208 F g^–1^ 1 A g^–1^	83.5% 5,000 cycles 10 A g^–1^	3 M KOH	[148]
Honeycomb	rGO/NiO/Co_3_O_4_	Microwave irradiation	910 F g^–1^ 20 mV s^–1^	89.9% 2,000 cycles 100 mV s^–1^	0.1 M KOH	[149]
Honeycomb	rGO/Co_2_SiO_4_	Hydrothermal	429 F g^–1^ 0.5 A g^–1^	92% 10,000 cycles NA	3 M KOH	[150]
Honeycomb	Ni_0.85_Se on NF	Hydrothermal	3,105 F g^–1^ 1 A g^–1^	90.1% 5,000 cycles 10 A g^–1^	3 M KOH	[151]
*Other animal-inspired structures*						
Beehive	porous carbon	Carbonization, activation	314 F g^–1^ 0.5 A g^–1^	96% 2,000 cycles 5 A g^–1^	6 M KOH	[94]
Beehive	NiFe_2_O_4_/ Ni nanocone on Ni foil	Electrodeposition	483 F g^–1^ 5 A g^–1^	95.3% 10,000 cycles NA	1 M KOH	[93]
web	V_3_O_7_ on carbon cloth	Hydrothermal	198 F g^–1^ 1 A g^–1^	∼97% 100,000 cycles 10 A g^–1^	1 M Na_2_SO_4_	[118]
Hedgehog	Ni–Mn oxide	Hydrothermal	1,016 F g^–1^ 0.5 A g^–1^	NA	6 m KOH	[121]
	Ni–Mn sulfide		1,430 F g^–1^ 0.5 A g^–1^	NA		
Hedgehog	NiCo_2_O_4_ @Ni_*x*_Co_*y*_MoO_4_	Two-step hydrothermal	861.3 C g^–1^ 1 A g^–1^	99.5% 10,000 cycles 5 A g^–1^	PVA-KOH	[119]
Whisker	PANI on carbon fibers	Chemical polymerization	427 F g^–1^ 5 mV s^–1^	90% 3,000 cycles 1 A g^–1^	1 M H_2_SO_4_	[123]
Whisker	Ni–Co hydroxides	Hydrothermal	918.9 F g^–1^ 0.2 A g^–1^	98.7% 3,000 cycles 2 A g^–1^	6 M KOH	[124]
Caterpillar	NiCo_2_S_4_ on NF	hydrothermal /sulfurization	1,777 F g^–1^ 1 A g^–1^	83% 3,000 cycles 10 A g^–1^	5 M KOH	[126]
Caterpillar	PANI/P_4_VP-g-GMWCNT	Chemical polymerization	1,065 F g^–1^ 0.5 A g^–1^	92.2% 1,000 cycles 1 A g^–1^	0.5 M Na_2_SO_4_	[127]
Worm	NiMoO_4_ on carbon nanofiber	Hydrothermal	1,088.5 F g^–1^ 1 A g^–1^	73.9% 5,000 cycles 10 A g^–1^	2 M KOH	[130]
Worm	Ni–Co–P on NF	Electroless electrolytic deposition	222.16 F g^–1^ 1 mV s^–1^	105% 1,000 cycles 50 mV s^–1^	6 M KOH	[132]
Worm	N,S–carbon	Carbonization	456 F g^–1^ 0.3 A g^–1^	NA	1 M H_2_SO_4_	[82]
Plume	Ni_3_S_2_ on rGO-NF	Hydrothermal	1462 F g^−1^ 1 A g^−1^	93.% 2,000 cycles 1 A g^–1^	2 M KOH	[133]
Nest	N, P-co-doped carbon	Microwave-assisted solvothermal method	171 F g^−1^ 0.2 A g^−1^	96.2% 5000 cycles 2 A g^–1^	6 M KOH	[83]
Nest	Fe:MnO_2_	Electrodeposition	273 F g^–1^ 5 mV s^–1^	92% 1,000 cycles 100 mV s^–1^	1 M Na_2_SO_4_	[135]
Nest	N-doped carbon–V_3_O_7_	Hydrothermal, in situ photopolymerization method	660.63 F g^–1^ 0.5 A g^–1^	80.47% 4,000 cycles 10 A g^–1^	1 M Na_2_SO_4_	[137]
Ant-nest	NiMoO_4_/carbonized melamine sponge	Carbonization/solvothermal	1,689 F g^–1^ 1 A g^–1^	86.7% 5,000 cycles 10 A g^–1^	3 M KOH	[140]
Ant-nest	MnO_2_/carbon	Annealing, simple mixing	662 F g^–1^ 1 A g^–1^	93.4% 5,000 cycles 1 A g^–1^	6 M KOH	[142]

#### Spider Web-Like Structure

**Fig. 3:**
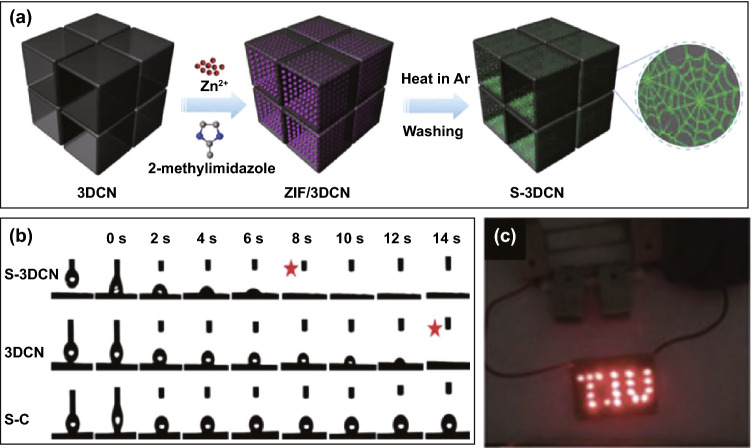
**a** The illustrated scheme of the synthesis process, **b** The results of water-drop experiments of S-3DCN, 3DCN, and S-C, and **c** LED light devices powered by three supercapacitors connected in series. Reproduced with permission from Ref. [110]. Copyright 2018 Elsevier

Nature has always motivated and inspired human being for the fabrication of interesting and attractive nanostructures-based electrode materials [105– 107]. Both materials properties and determination of architectures are important for variety of applications [108, 109]. Spider webs are commonly found anywhere – at parks or even at homes. Spider webs are made from silk known for its exceptional toughness and flexibility, and water resistivity. Inspired by these characteristics, Deng et al. [110, 111] prepared a special design structure for energy storage device with exceptional mass transfer abilities. Figure 3a shows the schematic diagram of the preparation of 3D carbon network (3DCN) with bionic surface using zeolitic imidazolate frameworks (ZIF) as precursors. ZIF-8 polyhedron on carbon surface were connected to each other, forming spider web network-like structure. After annealing at 800 °C in argon for 2 h, the desired structure was achieved by washing away ZIF-8 with HCl and retaining 3D carbon network with “spider web”-like carbon, S-3DCN. The detailed mass transfer abilities for all three samples are given in Fig. 3b. Herein, S-3DCN portrayed a full adsorption of water drop at 8 s, out-performing 3DCN (14 s) and S-C (> 14 s), depicting S-3DCN has the best transportation ability. The as-prepared electrode material was further considered for energy storage applications and the solid-state SSC device (S-3DCN//S-3DCN) was fabricated. The nature-inspired S-3DCN spider web-like microstructures show multiple active sites toward electrolyte, numerous pores, excellent wettability of electrolyte. The designed SSC device successfully illuminated 19 LEDs as shown in Fig. 3c, which confirmed the advantaged of S-3DCN materials toward multiple applications.

**Fig. 4:**
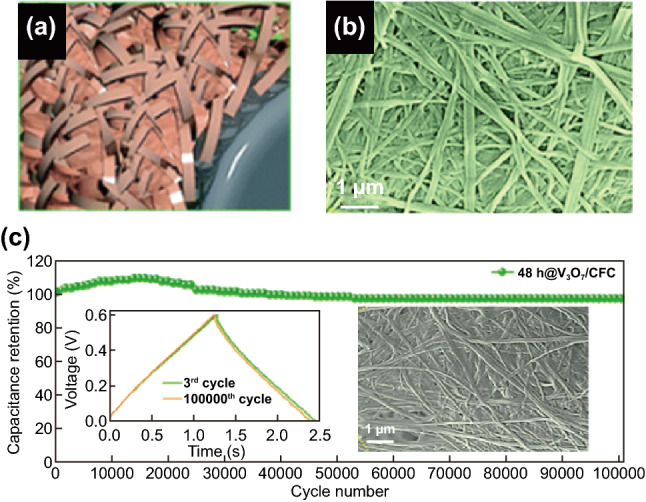
**a** Schematic representation of the in situ growth of V_3_O_7_ spider web-like nanowires, **b** SEM image V_3_O_7_/CFC substrates. **c** Cycling stability of V_3_O_7_/CFC SSCs devices at a constant current density of 10 A g^–1^ for 100,000 cycles. Reproduced with permission from Ref. [118]. Copyright 2018, Royal Society of Chemistry

The construction and design of 1D nanoarchitecture and the flexibility of binder-free electrode material have shown great interest for electrode fabrication. The practical capacitance and stability of the metal oxide greatly depend on the composition, synthesis condition, and morphology of the structure [43, 63, 112, 113, 114, 115, 116, 117]. Manikandan et al. [118] designed binder-free vanadium oxide spider web-like nanostructure by using facile in situ hydrothermal technique for SSC devices. Figure 4a-b shows the schematic diagram and the scanning electron microscopy (SEM) image of the as-synthesized spider web-like structure. The nature-inspired based SSC device exhibited exceptional stability of 97% after 100,000 cycles (Fig. 4c). Even after 100,000 cycles, the spider web-inspired nanostructure was preserved as shown in the inset of Fig. 4c.

#### Hedgehog Quills Structure

**Fig. 5:**
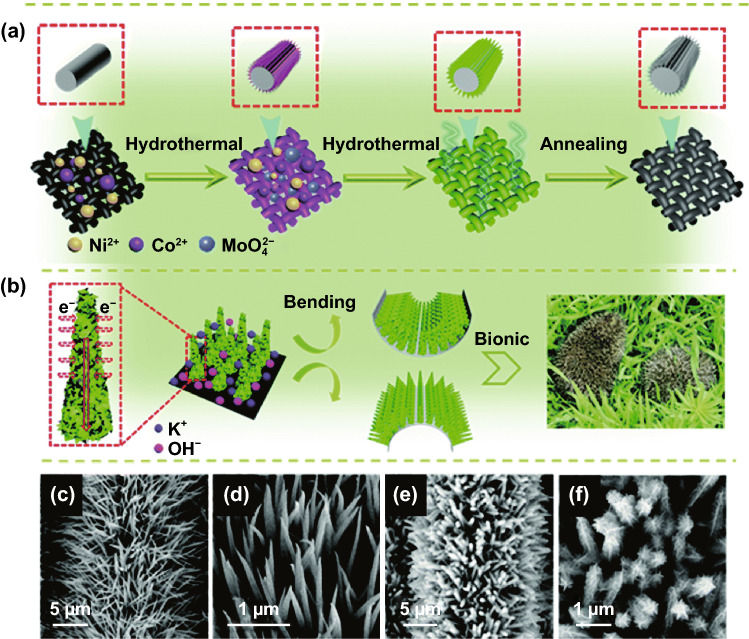
**a** Schematic illustrations of NiCo_2_O_4_@Ni_x_Co_y_MoO_4_ nanostructures (carbon fabric (black), NiCo_2_O_4_ nanoneedle clusters (pink), the NiCo_2_O_4_@Ni_x_Co_y_MoO_4_ precursor (green), NiCo_2_O_4_@Ni_x_Co_y_MoO_4_ (gray)); **b** charge transport and stress release in these hedgehog-like nanoneedle-cluster nanostructures; **c, d** SEM images of NiCo_2_O_4_ nanoneedle clusters; and **e, f** NiCo_2_O_4_@Ni_x_Co_y_MoO_4_ nanostructures. Reproduced with permission from Ref. [119]. Copyright 2018, Royal Society of Chemistry

Hedgehogs are unique pets that have spines consisting of hollow hairs. These spines, called as quills, can be curled, or straightened upon muscle control. Inspired by the curling up and relaxation of their quills and the exterior structure of hedgehogs, researchers have utilized these structures to cater an electrode’s architecture for SC application [119, 120, 121]. Sun et al. [119] reported of hedgehog-inspired electrode material for flexible SC devices. Figure 5a shows the preparation of NiCo_2_O_4_@Ni_x_Co_y_MoO_4_ core–shell hedgehog-like nanoneedle-clusters nanostructures. The synthesis was done in two-step facile hydrothermal method. In the first step, carbon fabric was used as a current collector and a substrate to grow NiCo_2_O_4_ hedgehog-like nanoneedles with a maximum diameter of 150 nm (Fig. 5c-d). In the second step, Ni_x_Co_y_MoO_4_ nanosheets were wrapped on the initially prepared NiCo_2_O_4_ nanoneedle clusters, forming NiCo_2_O_4_@Ni_x_Co_y_MoO_4_ core–shell hedgehog-like nanoneedle-cluster nanostructures (Fig. 5e-f). Figure 5b shows the utilization of the hedgehog-like structure for charge transport and stress release mechanism. Such combination of nanosheets grown on nanoneedles may be beneficial in giving sufficient space between the active materials which can provide better electrolyte infiltration and plentiful electroactive sites for redox reaction. The as-assembled all-solid-state flexible battery-type hybrid supercapacitor (HSC), NiCo_2_O_4_@Ni_x_Co_y_MoO_4_//AC, demonstrated outstanding outcomes with a high specific capacitance of 207 F g^–1^ (1 A g^–1^) a high energy density (64.7 Wh kg^–1^ at 749.6 W kg^–1^) and promising cycling stability (nearly 100% after 10,000 cycles).

#### Whisker-Like Structures

**Fig. 6:**
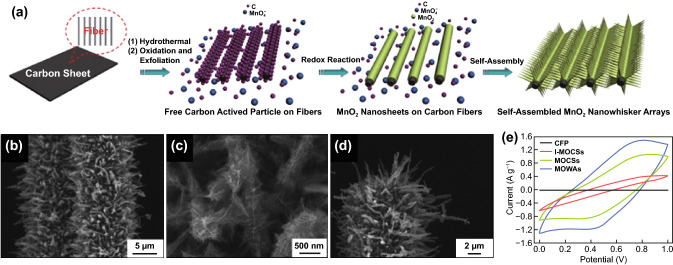
**a** Schematic illustration for the formation processes, (**b**–**d**) SEM images of the MOWAs, and **e** CV curves of the different electrode materials at 50 mV s^−1^. Reproduced with permission from Ref. [122]. Copyright 2012, Royal Society of Chemistry

Luo et al. [122] synthesized self-assembled whisker-like MnO_2_ arrays on carbon fiber paper (MOWAs) using simple in situ redox replacement reaction in a hydrothermal method (Fig. 6a). In their study, different amount (3–15 mM) of potassium permanganate (KMnO_4_) was used as precursor to MnO_2_ yielding to different morphologies. At 7 mM KMnO_4_, highly ordered whisker-like MnO_2_ arrays are consistently observed covering the whole carbon fiber (Fig. 6b). At a higher magnification (Fig. 6c), each whisker is made up of many interconnected and ultrathin nanosheets. An individual MOWA is shown in Fig. 6d portraying a length of 3–5 μm and about 0.5 μm in diameter at the middle section. Other morphologies were also observed upon changing KMnO_4_’s concentration to 3 mM (carbon fiber/MnO_2_ core–shell nanostructures, MOCSs) and 15 mM (ill-defined carbon fiber/MnO_2_ core–shell nanostructures, I-MOCSs). Figure 6e shows a comparative study of the cyclic voltammetry (CV) curves of carbon fiber paper (CFP), MOWAs, as well as the two other prepared structures, MOCs and I-MOCs. Among all electrodes, MOWAs exhibited the largest CV curve. The specific capacitance obtained for MOWAs electrode at 100 mA g^–1^ is 274.1 F g^–1^. The long-term cycling stability of MOWAs electrode resulted in a retention of 95% after 5,000 cycles (100 mA g^–1^). With such unique structure of small MnO_2_ sheets directly attached on CFP, MOWAs’ architecture provided well separated yet conductive sheets that paved way for better ion insertion and transport. Other whisker-like structured electrodes are reported in polyaniline on carbon nanofiber (CNF) [123], floss-like Ni–Co binary hydroxide composites assembled with whisker-like nanowires [124], and polyaniline (PANI) whiskers [125].

#### Caterpillar and Worm-Like Structures

**Fig. 7:**
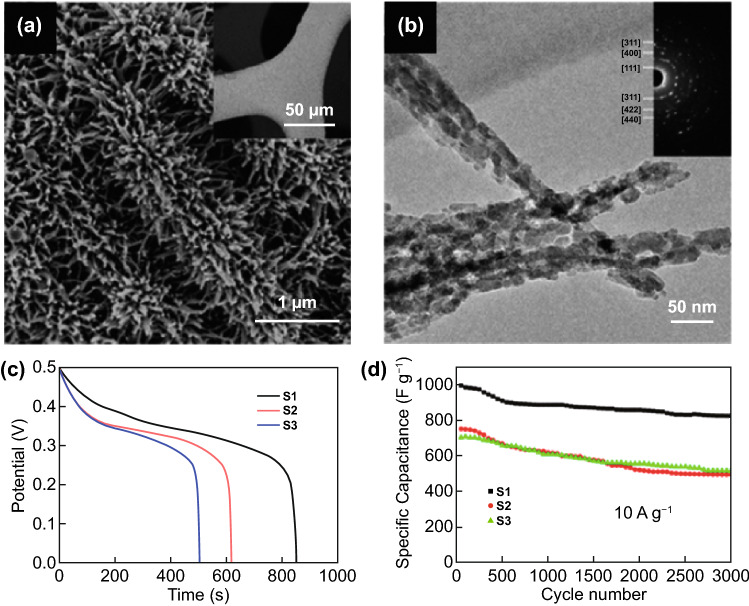
**a** SEM images of NiCo_2_S_4_/NF. **b** TEM image of nanowires from NiCo_2_S_4_ NSNWs; the inset in (b) shows SAED pattern, **c** GCD curves at 1 A g^–1^, and **d** Stability at 10 A g^–1^ of the S1, S2, and S3 electrodes. Reproduced with permission from Ref. [126]. Copyright 2017, ACS Publications

Caterpillars and worms are both cold-blooded species typically with a long tube-like body. However, worms consist of smooth-structured body that do not have legs, eyes nor hair. In contrast, caterpillars have segmented bodies which appears to be rough and hairy. In a nanostructure level, caterpillar-like and worm-like structures have been investigated in SC applications. For instance, caterpillar-like NiCo_2_S_4_ nanocrystal arrays on nanofibers (NF) [126] and polyaniline/CNT hybrids with core–shell structures [127] are successfully synthesized as electrode materials. Figure 7a shows the SEM image of the NiCo_2_S_4_ nanosheet@nanowires (NSNW) which exhibits a caterpillar-like structure [126]. The NiCo_2_O_4_ consists of vertical nanosheets aligned on NF with each sheet having multidirectional nanowires. Figure 7b shows a transmission electron microscopy (TEM) image of the nanowire with a dimension of bottom core ∼50 nm and a tip ∼30 nm. Aside from the caterpillar-like NiCo_2_S_4_ NSNW structures, other structures were prepared by varying the time of maintained reaction. The sealed autoclave maintained at 95 °C for 12, 10, and 8 h yielded the Ni–Co precursor NSNWs, Ni–Co precursor NSNP, and Ni–Co precursor NS, respectively. The difference in morphologies and its effect in electrochemical performance were evaluated and the discharge curves are shown in Fig. 7c. NiCo_2_S_4_ nanosheet@nanowires (NSNW, S1), NiCo_2_S_4_ nanosheet@nanoparticles (NSNP, S2), and NiCo_2_S_4_ porous nanosheets (NS, S3) and their specific capacitances are 1,777, 1,238, and 1,010 F g^–1^, respectively, at the same current density of 1 A g^–1^. The caterpillar-like structure also benefited the S1 electrode with a retention of 83% after 3,000 cycles (10 A g^–1^), compared with S2 (66%) and S3 (73%) (Fig. 7d).

**Fig. 8:**
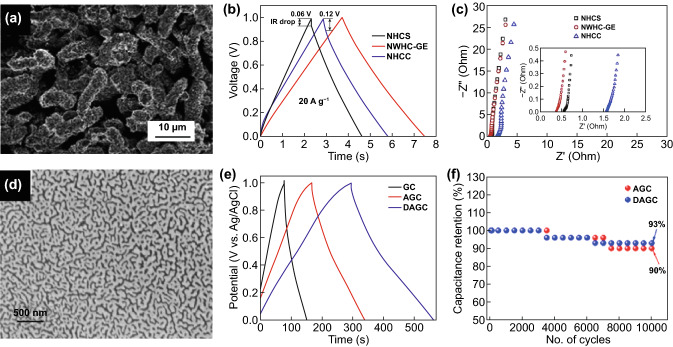
**a** SEM images of NWHC-GE before heat treatment; comparison of (**b**) GCD curves at 20 A g^–1^, and **c** Nyquist plot for different electrodes. Reproduced with permission from Ref. [128]. Copyright 2017, Elsevier. **d** SEM image of DAGC sheets; **e** comparison of (b) GCD curves at 1 A g^–1^, and **f** Stability test for different electrodes. Reproduced with permission from Ref. [82]. Copyright 2020, Elsevier

Worm-like structures have been prepared in N-doped graphitized porous carbon [128], mesoporous carbon [129], NiMoO_4_ coaxially decorated on electro-spun CNF [130], amorphous MnO_2_ nanowires grown on textiles [131], Ni–Co–P deposited on NF [132], and N/S-co-doped porous carbon [82]. Figure 8a shows the SEM image of a nitrogen-doped worm-like hierarchical porous carbon with graphitized porous carbon embossment (NWHC-GE), which was prepared by polymerization-induced colloid aggregation method followed by coordination–pyrolysis process [128]. The worm-like structure was likely plausible due to the presence of ferrous sulfate heptahydrate (FSH) as a precursor. Aside from the worm-like structure formed from the addition of 0.01 mol FSH in the material preparation, the key influence of FSH leads to formation of other structures, such as N-doped hollow carbon sphere (NHCS, no FSH was added) and N-doped hollow carbon capsule (NHCC, 0.02 mol FSH was added). Figure 8b shows a comparative GCD profiles of NHCS, NWHC-GE, and NHCC at a current density of 20 A g^–1^ portraying the longest GCD curved favorable to NWHC-GE. At 1 A g^–1^, the higher specific capacitance of NWHC-GE is 178 F g^–1^is attained compared to NHCS (114 F g^–1^) and NHCC (156 F g^–1^). A better electrical conductivity and lower resistivity were also observed for NWHC-GE (0.39 Ω) than that of NHCS (0.57 Ω) and NHCC (1.58 Ω) (Fig. 8c). Such improved performance is credited to a higher graphitization degree and an optimized nitrogen-doping content for NWHC-GE.

Similarly, Gopalakrishnan et al. [82] reported worm-like hierarchical structures based on nitrogen, sulfur-co-doped porous carbon were derived from ginger. Inspired by ginger as biomass source for carbon, ginger was pre-activated using NaCl/KCl followed by carbonization (800 °C) and removal of salt ions through washing with diluted HCl (product denoted as AGC). To dope AGC with nitrogen and sulfur, thiourea was used as precursor and went under another carbonization (800 °C) (product denoted as DAGC). The final structure of the doped & activated ginger carbon (DAGC) with unique worm-like pore structure and interconnected cavities is shown in Fig. 8d. The worm-like structure not only gained a high specific surface area (720 m^2^ g^–1^) but also yielded to an improved electrochemical performance. Figure 8e shows a comparative GCD curves for DAGC, AGC (activated ginger-derived carbon), and GC (ginger-derived carbon without any activation and doping). Clearly, DAGC showed a lengthy charge and discharge profiles which can be acquainted to a better ion storage and high capacitive performance. The highest performance conducted at 1 A g^–1^ was obtained for DAGC (268 F g^–1^) compared to GC (75 F g^–1^) and AGC (172 F g^–1^). The stability for GC and DAGC were compared at constant current density (Fig. 8f). The superior electrochemical performance can be acquainted to DAGC’s thin carbon nanosheets morphology with worm-like pore structures and heteroatom doping, which utilized rapid ion transfers and maximum charge storage capacity [73, 74, 75].

#### Plume-Like Structure

**Fig. 9:**
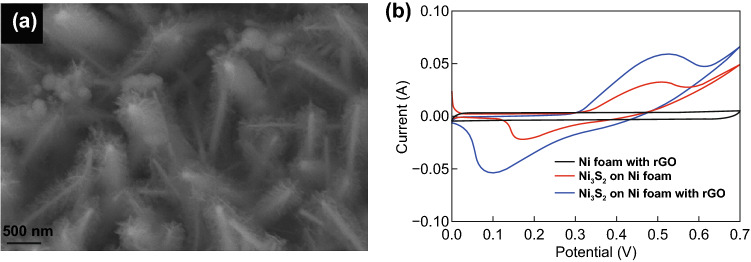
**a** SEM image plume-like structure and **b** comparison of CV curves plume-like structure compared to rGO on NF and Ni_3_S_2_. Reproduced with permission from Ref. [133]. Copyright 2017, Elsevier

A plume is similar to a structure of a bird’s feather that consists of tiny hair-like strands. Jinlong et al. [133] initially deposited graphene oxide (GO) sheets on NF by dipping in a GO dispersion and thermal reduction annealing. Then, Ni_3_S_2_ was grown by hydrothermal method on the pre-deposited GO on NF. The final resulting structure is a plume-like Ni_3_S_2_ grown on the NF with thermal reduced graphene oxide (rGO) architecture as shown in Fig. 9a. Figure 9b shows the CV curve representing the electrochemical performance of the plume-like structure compared to rGO on NF and Ni_3_S_2_ on NF only. At a scan rate of 2 mV s^−1^, larger non-rectangular CV curves were observed for the Ni_3_S_2_ on NF with rGO electrode. The plume-like structured electrode delivered a high capacitance of 1,462 F g^−1^ (1 A g^−1^) and retained 98.34% of its capacitance on the first 1,000 cycles. The plume-like Ni_3_S_2_ on NF with rGO exhibited better SC performance and improved cycling stability which is valuable for energy storage applications.

#### Nest-Like Structures

**Fig. 10:**
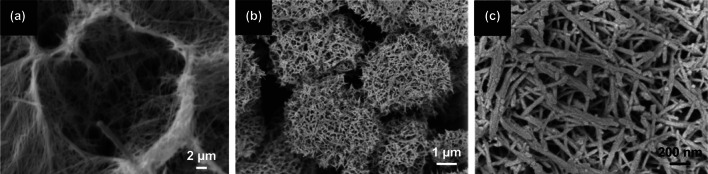
**a** SEM image of nest-like The Ni@Ni_1.4_Co_1.6_S_2_. Reproduced with permission from Ref. [138]. Copyright 2015, Royal Society of Chemistry. **b-c** SEM image of nest-like structures based from MnO_2_. Reproduced with permission from Ref. [139]. Copyright 2013, Elsevier

By nature, nests are built by animals, such as birds, to hold their eggs and to serve as a home for the young ones. Bird’s nest comprises of dried leaves, branch, or grasses that is coiled into a cup-shape. Nest-like architectures are studied and structured in electrode materials, such as N- and P-co-doped mesoporous carbon [83], glucose-derived nitrogen-doped hollow carbon [134], Fe-doped MnO_2_ [135], polyaniline [136], and V_3_O_7_ [137]. A nest-like structure based on Ni@Ni_1.4_Co_1.6_S_2_ [138], MnO, and V_3_O_7_ [137] are shown in Fig. 10. Mi et al. [138] initially synthesized Ni@Ni_3_S_2_ with a nest-like structure. Ni@Ni_1.4_Co_1.6_S_2_ was then synthesized by a Co-exchange method by using Ni@Ni_3_S_2_ as a template. The Ni@Ni_1.4_Co_1.6_S_2_ is composed of a network of nanowires which forms numerous micro-/nanoholes mimicking a nest (Fig. 10a). With a similar structure yet with the addition of Co-ions, the specific capacitance of Ni@Ni_1.4_Co_1.6_S_2_ is 122 F g^–1^, compared to Ni@Ni_3_S_2_ (89 F g^–1^) at 1 A g^–1^. Figure 10b shows a bird’s nest-like structures based on MnO_2_ [139]. The self-organized structure formed clusters with ~ 4–5 μm diameter consisting of interconnected nanowires (Fig. 10c). With such organized structure, the maximum specific capacitance of 917 F g^–1^ at a current density of 5 mA cm^–2^ was obtained.

**Fig. 11:**
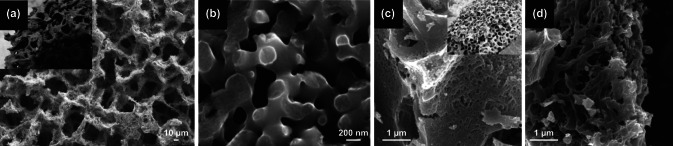
**a** SEM image of ant-nest-like NiMoO_4_/CMS, inset is ant-nest. Reproduced with permission from Ref. [140]. Copyright 2018, Royal Society of Chemistry. **b** SEM image of carbon‐based ant-nest-like structures. Reproduced with permission from Ref. [141]. Copyright 2019, Wiley. **c** SEM image of ant-nest-like ANHPC and **d** SEM image MnO_2_/ANHPC. Reproduced with permission from Ref. [142]. Copyright 2018, ACS Publications

Another nest-like structure is based on the home of ants or anthills. Anthills are commonly built underground. Inside an anthill is a massive network of interior channels and chambers. Inspired by such unique interconnected structure, ant-nest-based nanostructures have been utilized as electrode materials. A unique ant-nest-like structured electrode was prepared in NiMoO_4_/ carbonized melamine sponge (CMS) using solvothermal reaction (Fig. 11a) [140]. NiMoO_4_ nanorods are grown on the CMS by solvothermal reaction while maintaining the interconnected channels of CMS sponge. The optimized NiMoO_4_/CMS electrode exhibited a high specific capacitance of 1,689 F g^–1^ (1 A g^–1^). Miao et al. [141] studied carbon‐based ant-nest-like structures are prepared using NF supported hierarchical porous carbon (NF-HPC). A 3D crossed‐linked and associated backbones (diameter of ≈200 nm) aided the formation of highly uniform and well‐interconnected porous structure (Fig. 11b). As a symmetric SC device, the outstanding electrochemical performance of the device resulted in a high specific capacitance (292 at 0.25 A g^–1^) and long‐term cycling stability (100% at 5 A g^–1^ after 30,000 cycles). Lastly, a unique 3D ant-nest-like hierarchical porous carbon (ANHPC) is shown in Fig. 11c [142]. The ant-nest-like structure of ANHPC possesses large surface area (2,372 m^2^ g^–1^) and high pore volume (1.936 m^3^ g^–1^. The exceptional structure was then utilized to embed MnO_2_ and obtain MnO_2_/ANHPC composites (Fig. 11d). It is clearly observed that the carbon skeleton structure has not collapsed and retained the structure of ANHPC. The ant-nest-inspired structures give a favorable architecture for rapid ion transfer/diffusion. The combination of EDLC-based ANHPC and pseudocapacitance-based MnO_2_ yielded to a high specific capacitance of 662 F g^–1^ at 1 A g^–1^ compared to ANHPC at 254 F g^–1^. The detailed electrochemical performance of animal-inspired structure tabulated in Table 2.

### Human Body-Inspired Structures

#### Spine-Like Structure

**Fig. 12:**
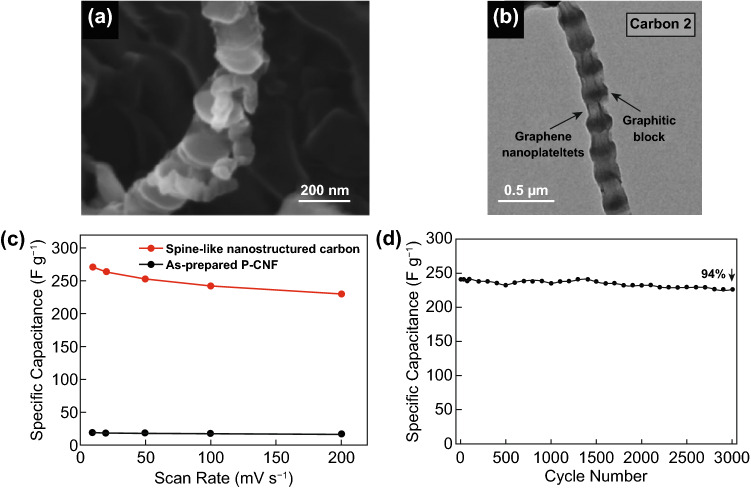
**a-b** SEM and TEM of spine-like nanostructured carbon, **c** rate capabilities of as-prepared P-CNF and spine-like nanostructured carbon, **d** cycle stability of the spine-like nanostructured carbon measured at a scan rate of 100 mV s^–1^. Reproduced with permission from Ref. [84]. Copyright 2014, Nature publisher

Park et al. [84] prepared spine-like nanostructured carbon interconnected by graphene with SC applications. The preparation of spine-like graphene-interconnected nanostructured carbon consists of three steps: i) preparation of platelet-type CNF (P-CNF) by chemical vapor deposition (CVD), ii) an expanding process by oxidation treatment, and iii) a co-solvent exfoliation method and reduction processes. Figure 12a-b shows the SEM and TEM image of the spine-like nanostructured carbon which is composed of regularly occurring intervals of exfoliated graphitic blocks and graphene nanoplatelets. In a three-electrode system, the spine-like nanostructured carbon exhibited significantly improved electrochemical performance (272 F g^–1^ at 10 mV s^–1^) compared to as-prepared P-CNF (19 F g^–1^) (Fig. 12c). To further use into practical application, the two-electrode system delivered a high capacitance (238.8 F g^–1^ at 2.5 A g^–1^), rate capability (230 F g^–1^ at 200 mV s^–1^, above 85% of the initial value at 10 mV s^–1^), and cycle stability (94% after 3,000 cycles) for the spine-like nanostructured carbon (Fig. 12d).

#### Finger-Like Structure

**Fig. 13:**
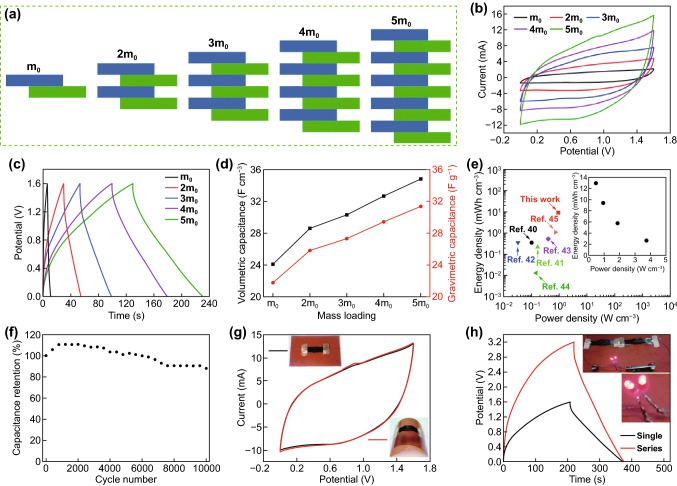
Electrochemical performances and applications of VFASCs. **a** Schematic diagram, **b** CV curves at a scan rate of 20 mV s^−1^, **c** GCD curves, and **d** volumetric capacitances and gravimetric capacitances with various mass loadings of the VFASCs. **e** Energy and power density plot and **f** cycle life of the VFASCs with a mass loading of 5*m*_0_. **g** CV curves of the finger-like ASCs in the straight and bent states. **h** GCD curves of a single and two finger-like supercapacitors connected in series. The inset shows two lit LED lamps powered by the two VFASCs in series. Reproduced with permission from Ref. [158]. Copyright 2017, Royal Society of Chemistry

Finger-like structures are used as effective designs for electrode materials in multifunctional integrated micro/nano systems [152, 153, 154, 155, 156]. In‐plane finger-like structures are advantageous for micro‐supercapacitors for it provides suitable accessibility for ion transport as the edges of the active electrodes are exposed to the electrolyte. Also, the finger-like design eliminates the use of separators as needed in conventional sandwich structures of SCs which also decreases the resistance and leads to high-frequency response as the distance between the electrode finger arrays is small [157]. Wang et al. [158] fabricated vertical finger-like asymmetric supercapacitors (VFASCs) comprising of rGO–manganese dioxide–polypyrrole (rGO-MnO_2_-PPy) as positive electrode and RGO–molybdenum trioxide (rGO–MoO_3_) as negative electrode. Various mass loading was investigated with structures mimicking 2 to 10 finger-like electrodes (Fig. 13a). The CV (Fig. 13b) and GCD (Fig. 13c) curves showed the highest performance for the 10 finger-like electrodes (5m_0_). The specific capacitance increased with the increased of mass loading (Fig. 13d) with 5m_0_ recording the highest capacitance of 31.4 F g^–1^ (34.8 F cm^−3^). The 5m_0_ electrode showed a high energy density of 12.94 mW h cm^−3^ (power density at 0.47 W cm^−3^) and still maintained the high value of 2.59 mW h cm^−3^ (power density at 3.72 W cm^−3^) with 88.2% capacitance retained after 10,000 cycles (Fig. 13e-f). For practical application, bending experiment was done to show the flexibility of the electrode as shown in Fig. 13g. Moreover, two electrodes connected in series accumulating 3.2 V were successful in lighting two LEDs (Fig. 13h).

#### DNA-Like Structure

**Fig. 14:**
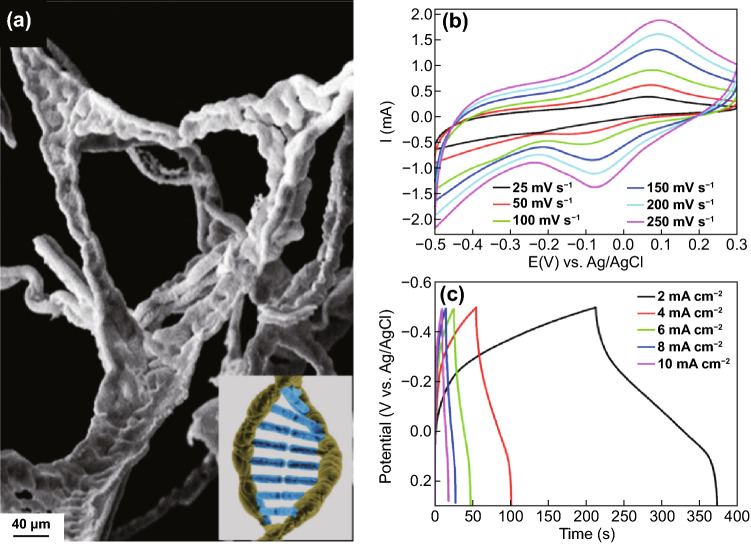
**a** SEM image depicting DNA-like double helical tripodal microfiber superstructure along with representative DNA image shown in the inset for comparison. **b** Scan rate-dependent CV curves and **c** GCD curves at different current densities for DNA-like double helical WO_3__–x_/C microfibers. Reproduced with permission from Ref. [159]. Copyright 2020, ACS Publications

Another fascinating nature-inspired nanoarchitecture is a double helical DNA-like WO_3__–x_/C microfiber superstructure [159]. Salkar et al. [159] prepared a self-assembly of in situ carbon fiber encapsulated by WO_3__–x_/C nanorods depicting a DNA-like structure as shown in Fig. 14a. The double helical DNA-inspired assembly provides favored structure allowing better participation of ions during electrochemical reaction. Figure 14b shows the CV curves with different scan rate (25 to 250 mV s^–1^) across the − 0.5 to 0.3 V potential range. At 25 mV s^–1^, the specific capacitance is 169.2 F g^–1^. Using the GCD curves, the highest specific capacitance was recorded at 498.4 F g^–1^ at 1.2 A g^–1^ (equivalent to areal capacitance of 401.4 mF cm^–2^ at 2 mA cm^−2^) (Fig. 14c). A solid-state asymmetric supercapacitor (ASC) device was assembled a deliver a power density of 498 W kg^–1^ at an energy density of 15.4 Wh kg^–1^. The rare DNA-like morphology only justified its unusual yet important structure in the development of electrode nanoarchitectures.

#### Dendrite-Like Structure

**Fig. 15:**
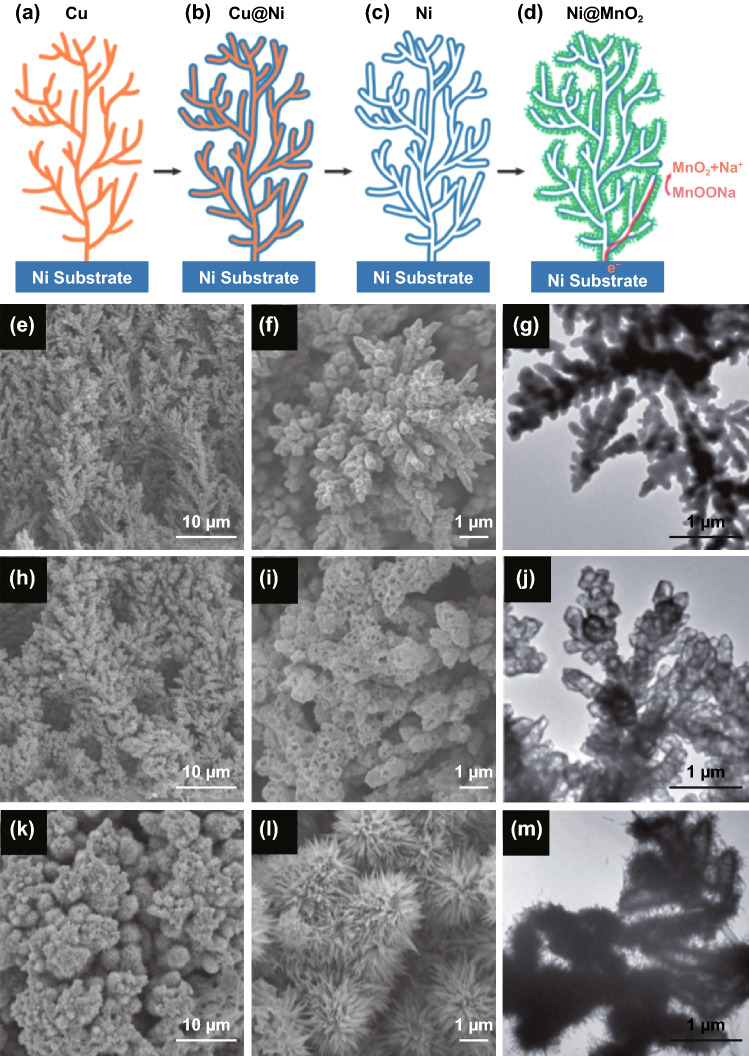
Synthesis of MnO_2_ nanowires supported on hollow Ni dendrites. **a** Electrodeposition of a nanoforest of Cu dendrites; **b** electroplating of Ni on Cu dendrites; **c** selective dissolution of Cu; **d** electrodeposition of MnO_2_ nanowires on hollow Ni dendrites to form a hierarchical Ni@MnO_2_ porous structure. SEM and TEM images of (**e**–**g**) Cu dendrites, **h**–**j** hollow Ni dendrites, and **k**–**m** Ni@MnO_2_ structure, respectively. Reproduced with permission from Ref. [163]. Copyright 2013, Royal Society of Chemistry

Dendrites are pronged extensions of a nerve cell which is similar to a tree-like structures. Dendrite formation has also been observed in mineral crystal growth, as well as, in snowflake and frost pattern formations. These unique structures have been observed in the growth of Au dendrites containing long back bone stems with several branches and highly corrugated structures [160], Co_3_O_4_ nanostructure made up of nanorods [161], and dendrite-like MnO_2_ nanostructures grown on carbon cloth [162]. Figure 15 shows the synthesis of MnO_2_ nanowires grown on hollow Ni dendrites prepared by Sun et al. [163]. Initially, Cu dendrites were first prepared on Ni substrate using electrodeposition (Fig. 15a, e–g). The as-formed Cu dendrites were then coated with a thin layer of Ni using electroplating (Cu@Ni) (Fig. 15b). Then, Cu was selectively removed from Cu@Ni through anodic dissolution, leaving a hollow Ni (Fig. 15c, h-j). Finally, Ni@MnO_2_ was prepared by growing MnO_2_ nanowires on the surface of hollow Ni using anodic pulse electrochemical deposition (Fig. 15d, k-m). When applied as an electrode, the Ni@MnO_2_ electrode delivered a specific capacitance of 1125 F g^–1^ (5 mV s^–1^) at a MnO_2_ mass loading of 0.35 mg cm^–2^. When a higher MnO_2_ mass loading was increased to 1.8 mg cm^–2^, the specific capacitance resulted in 303 F g^–1^ (5 mV s^–1^). The outstanding electrochemical performance of the Ni@MnO_2_ electrode can be attributed to the following nanoarchitecture: first, the highly conductive hollow Ni dendrites acted as both support and current collector that allowed the pathway for fast electron transport; second, the MnO_2_ nanowire arrays permitted productive material utilization; lastly, the existence of hierarchical porous channels in the overall construction facilitates fast diffusion between the electrode and electrolyte [39, 40, 47, 48, 56, 164, 165]. The detailed electrochemical performance of human-inspired structure tabulated in Table 3.

**Table 3:** Comparison of human-inspired structures in three-electrode measurements

Electrode structure	Electrode materials	Method	Three-electrode measurements			Refs.
			Capacitance	Cycling stability	Electrolyte	
*Human body-inspired structures*						
Spine	Graphitic carbon	Co-solvent exfoliation	272 F g^−1^ 10 mV s^−1^	94% 3,000 cycles 100 mV s^−1^	1 M H_2_SO_4_	[84]
Finger	MoS_2_	Spray painting, laser patterning	8 mF cm^−2^ or 178 F cm^−3^ 10 mV s^−1^	~ 92% 1,000 cycles 0.22 A m^−2^	1 M NaOH	[152]
Finger	MXene-MoS film	Laser engraving	75.5 F g^−1^ or 173.6 F cm^−3^ 1 mV s^−1^	98% 6,000 cycles 0.335 A g^−1^	2 M ZnSO_4_	[153]
DNA	WO_3–*x*_/carbon Fiber	Simple mixing/reflux	401 mF cm^–2^ 2 mA cm^–2^	> 94% 5,000 cycles 8 mA cm^–2^	0.5 M H_2_SO_4_	[159]
Dendrite	Co_3_O_4_	Hydrothermal	207.8 F g^−1^ 0.5 A g^−1^	97.5% 1,000 cycles 1.8 A g^−1^	3 M KOH	[161]
Dendrite	MnO_2_ on carbon fiber cloth	Hydrothermal	430 F g^−1^ 1 A g^−1^	82.75% 10,000 cycles 1 A g^−1^	1 M Na_2_SO_4_	[162]
Dendritic	Ni@MnO_2_	Electrodeposition	1125 F g^−1^ 5 mV s^−1^	103% 1,000 cycles 12.5 A g^−1^	1 M Na_2_SO_4_	[163]

## Future Outlooks and Conclusion

In this review article, we have highlighted the importance of animal and human body-inspired materials down to the nanoscale level for SC application. Materials with different dimensionalities can be fabricated, tailored, and exploited according to several factors to construct nature-inspired formation of interconnected and hierarchical nanostructures. It is interesting to note that such dimensional structures can generate ordered structures, which are highly valued as the electrode materials. Still, the overall electrochemical performance will matter for practical use. Though having a high capacitance and capacitive property is highly desirable for many electrodes that has been researched, it is only one of the important properties that must be considered in constructing electrode materials. Nevertheless, a lot of hindrances have been assessed why scientifically studied electrodes cannot be launched in the market.

Nature-inspired materials with high porosity are found to be feasible for energy storage applications. The current article systematically summarized SC application of few of such nature-inspired materials. However, such materials also shave some drawbacks, which are enlisted below with future research directions.

1. Large scale production of such nature-inspired materials with cost-effectiveness is quite complicated. For example, metal precursors displayed considerable electrical and chemical properties. However, researchers must consider the long-term availability of these precious metals as well as their costs if they want to concentrate on producing metal-based electrode materials in commercial scale. Moreover, precise production of such material is also highly challenging. In this aspect, the 3D printing techniques have the ability to copy the natural structures. The printed products are also found to be highly flexible, which can be applicable for constructing flexible and stretchable supercapacitors. Therefore, future manufacturing of nature-inspired materials can be focused on these techniques for scalable production.
2. Apart from their structures and morphologies, other factors, like the nature of electrode materials, choice of electrolytes, use of binders, and nature of current collections, have also played significant role on the electrochemical performance of any electrode materials. Smart combination of EDLC-type and pseudocapacitive-type materials with nature-inspired structures is found to be a feasible strategy to improve the capacitive performance. Although, such combination requires much more research attention for commercialization.
3. In-depth knowledge of the charge storage mechanism of such nature-inspired materials is highly required for future research. In this aspect, the in situ characterization techniques like in situ TEM, in situ XRD, in situ Raman spectra, in situ XPS, etc. can be a pivotal approach to incorporate in material characterization in order to better understand the physiochemical properties of the active materials.
4. It is evident that the electrochemical performance of such nature-inspired materials can be tuned by manipulating the interfacial interactions of individual components. However, detailed theoretical study is necessary in this topic.
5. Nature-inspired structures based on newly developed 2D materials like MXene should be explored for a wide variety of applications.
6. The differences in EDLC-, pseudocapacitive-, and battery-type electrode materials; symmetric, asymmetric, and hybrid SC devices; and the appropriate selection of potential/voltage window as well as suitable equations for the calculations of energy density should be carefully selected as previously discussed [21, 166, 167].
